# Essential Amino Acid Enrichment and Positive Selection Highlight Endosymbiont's Role in a Global Virus-Vectoring Pest

**DOI:** 10.1128/mSystems.01048-20

**Published:** 2021-02-02

**Authors:** Kaitlyn N. Myers, Daniel Conn, Amanda M. V. Brown

**Affiliations:** a Department of Biological Sciences, Texas Tech University, Lubbock, Texas, USA; Cornell University

**Keywords:** amino acid biosynthesis, comparative genomics, genome-wide association study, mutualism, nutritional symbiont, plant-parasitic nematode, population genomics, symbiosis

## Abstract

Host-associated microbes display remarkable convergence in genome repertoire resulting from selection to supplement missing host functions. Nutritional supplementation has been proposed in the verrucomicrobial endosymbiont *Xiphinematobacter* sp., which lives within a globally widespread group of plant-parasitic nematodes that vector damaging nepoviruses to plants. Only one genome sequence has been published from this symbiont, leaving unanswered questions about its diversity, host range, role, and selective pressures within its hosts. Because its hosts are exceptionally resistant to culturing, this symbiont is best studied through advanced genomic approaches. To analyze the role of *Xiphinematobacter* sp. in its host, sequencing was performed on nematode communities, and then genomes were extracted for comparative genomics, gene ontology enrichment tests, polymorphism analysis, de Bruijn-based genome-wide association studies, and tests of pathway- and site-specific selection on genes predicted play a role in the symbiosis. Results showed a closely clustered set of *Xiphinematobacter* isolates with reduced genomes of ∼917 kbp, for which a new species was proposed. Symbionts shared only 2.3% of genes with outgroup *Verrucomicrobia*, but comparative analyses showed high conservation of all 10 essential amino acid (EAA) biosynthesis pathways plus several vitamin pathways. These findings were supported by gene ontology enrichment tests and high polymorphisms in these pathways compared with background. Genome-wide association analysis confirmed high between-species fixation of alleles with significant functional enrichment for EAA and thiamine synthesis. Strong positive selection was detected on sites within these pathways, despite several being under increased purifying selection. Together, these results suggest that supplementation of EAAs missing in the host diet may drive this widespread symbiosis.

**IMPORTANCE** *Xiphinematobacter* spp. are distinctly evolved intracellular symbionts in the phylum *Verrucomicrobia*, which includes the important human gut-associated microbe Akkermansia muciniphila and many highly abundant free-living soil microbes. Like *Akkermansia* sp., *Xiphinematobacter* sp. is obligately associated with the gut of its hosts, which in this case consists of a group of plant-parasitic nematodes that are among the top 10 most destructive species to global agriculture, by vectoring plant viruses. This study examined the hypothesis that the key to this symbiont’s stable evolutionary association with its host is through provisioning nutrients that its host cannot make that may be lacking in the nematode’s plant phloem diet, such as essential amino acids and several vitamins. The significance of our research is in demonstrating, using population genomics, the signatures of selective pressure on these hypothesized roles to ultimately learn how this independently evolved symbiont functionally mirrors symbionts of phloem-feeding insects.

## Abstract (precis)

*Xiphinematobacter* spp. are distinctly evolved intracellular symbionts in the phylum *Verrucomicrobia*, which includes the important human gut-associated microbe Akkermansia muciniphila and many highly abundant free-living soil microbes. Like *Akkermansia* sp., *Xiphinematobacter* sp. is obligately associated with the gut of its hosts, which in this case consists of a group of plant-parasitic nematodes that are among the top 10 most destructive species to global agriculture, by vectoring plant viruses.

## INTRODUCTION

Microbes often play critical roles in plant-feeding organisms (1), such as breaking down plant starches or supplementing essential amino acids (EAAs) for phloem-feeding insects (2, 3). Microbes have converged evolutionarily on this nutritional supplementation role many times across the bacterial tree of life, displaying reduction in costly and unnecessary metabolic processes except those necessary to maintain basic cellular processes and key symbiotic contributions (2, 4–6). While endosymbionts of plant-feeding organisms living above ground have been a major research focus, symbionts in plant feeders below ground have been poorly studied. One remarkable underground endosymbiont is *Xiphinematobacter* sp. (*Verrucomicrobia*), which lives within a globally widespread group of plant-parasitic nematodes. While this ancient symbiosis is not well understood, it has been proposed that the endosymbiont may have evolved to supplement essential amino acids missing in its host diet, mirroring symbionts of phloem-feeding Hemiptera (7, 8). This endosymbiont undergoes tight coevolution with its hosts (9–11) and has a degenerate genome with disproportionate conservation of essential amino acid biosynthesis genes (8) suggestive of a mutualistic role. Yet, to date, there is only one published genome study from this globally distributed symbiont. By investigating additional *Xiphinematobacter* genomes using population-level and species-level comparative genomics, the present study seeks to better understand the biological role of this symbiont in the evolutionary context of this symbiosis.

*Xiphinematobacter* sp. is of interest not only for convergent symbiosis evolution (7) but also as a contributor to the success of one of the most damaging plant-parasitic nematodes in agriculture (10, 12, 13). Broadly, nematodes may be the most numerically abundant animals on Earth (14) with millions of individuals per m^2^ of soil (15–17) that perform ecosystem services, such as nutrient cycling (18–20). Plant-parasitic nematodes make up a large portion of the community close to roots, causing up to 25% of global crop yield loss at costs of ∼$100 billion annually (21). Dagger nematodes (*Xiphinema* spp.) which host *Xiphinematobacter* sp. are among the top 10 most damaging nematodes as vectors of at least 13 nepoviruses, including tobacco ringspot and tomato ringspot virus (12, 13). *Xiphinematobacter* endosymbionts occur within the *Xiphinema americanum* species-complex, which includes up to 61 described species (22, 23) that infect an enormous range of host plants globally, including grape, almond, apricot, peach, nectarine, plum, prune, walnut, cherry, strawberries, soybeans, perennial orchards, and wild plants, including spruce, pine, and common weeds and grasses (24–26), sometimes at densities of 500 individuals per 250 cm^3^ of soil (27). Thus, given the host abundance of *Xiphinematobacter* sp., the symbiont’s high titer in its host (28), and its global distribution in soils (29, 30), *Xiphinematobacter* sp. appears to be a globally abundant endosymbiont.

Experimental research on the *Xiphinema*-*Xiphinematobacter* symbiosis remains exceedingly difficult, due to the difficulty of manipulating and experimenting with these nematodes under laboratory conditions (13, 24, 31, 32). Thus, in the present study, we sought to uncover field-sampled genomes of *Xiphinematobacter* sp. that could be analyzed using comparative genomics, single nucleotide polymorphism (SNP) analysis and population genomics using de Bruijn-based genome-wide association (DBGWAS) (33), and tests of site-wise natural selection in genes or pathways hypothesized to be important in the symbiont’s role to better understand the potential nutritional supplementation role of *Xiphinematobacter* sp. While PCR- and Sanger sequencing-based approaches have been helpful for identifying potential diversity and coevolution of *Xiphinematobacter* spp. with their hosts (9, 11, 34, 35), we focus on deeper analyses using comparative genomics (8, 36, 37) and microbial population genomics approaches (38–42) which are increasingly valuable tools to use to discover microbial function (43). We combine these approaches with community genome skimming, which is a promising new approach to simultaneously access plant-parasitic nematode hosts and their microbiomes (28).

Results showed that skimming was highly effective for yielding insights on nematode communities while producing full-length *Xiphinematobacter* genomes for which we propose a new species. Detailed comparative genomics, gene ontology (GO) enrichment analysis, population genomics, genome-wide association (GWAS), and tests for selection demonstrate distinct dynamics for essential amino acid and thiamine biosynthesis pathways, supporting them as nutrients that could be provisioned from symbiont to host.

## RESULTS

### *Xiphinematobacter* and *Xiphinema* spp. occurrence in field sample sites.

*Xiphinematobacter* spp. or *Xiphinema* spp. were detected at 11 sampled sites in Texas and New Mexico, as distant as 1,160 km apart. Six sites were found to have *Xiphinematobacter* spp., including samples from farms and mixed natural grass and shrub communities (see Table S1 in the supplemental material). There were up to 60 nematode species per 100-g sample, based on top cytochrome oxidase I (COI) blastn matches. There were 29 *Xiphinema* species (based on DNA fragment identities) detected overall, across samples based on COI matches. They included up to 9 species of *Xiphinema* per sample (see Table S2 in the supplemental material). Assembled nematode mitochondrial contigs include 16 contigs of >12,000 bp, comprising nearly full-length mitogenomes. Using both lower and higher kmer ranges during assembly yielded several long *Xiphinematobacter* contigs that comprised 50% to 95% of the bacterial genome at coverage depths ranging from 23.45× to 84.22× (Table S2), with assembly *N*_50_ values ranging from 16,926 to 871,420 bp. Postassembly contig alignment and reassembly produced full-length or nearly full-length genome sequences.

10.1128/mSystems.01048-20.5 TABLE S1 Collection sites with *Xiphinema* spp. Asterisks indicate sites with *Xiphinematobacter* spp. Download Table S1, DOCX file, 0.01 MB. Copyright © 2021 Myers et al. 2021 Myers et al. This content is distributed under the terms of the Creative Commons Attribution 4.0 International license.

10.1128/mSystems.01048-20.6 TABLE S2 Sequence and assembly data, including raw reads per bulk sample; number of *Xiphinema* spp. identified based on COI sequence; and assembly statistics for contigs matching *Xiphinematobacter* spp. Download Table S2, DOCX file, 0.01 MB. Copyright © 2021 Myers et al. 2021 Myers et al. This content is distributed under the terms of the Creative Commons Attribution 4.0 International license.

### Genome features of new *Xiphinematobacter* isolates.

Five of six *Xiphinematobacter* genomes appear complete based on gene repertoire, rRNA and tRNA completeness, and genome length, whereas the sixth genome appeared to be ∼80% complete (Table 1). For the *Xiphinematobacter* isolates sequenced in the current study with complete genomes (samples P15, P18, P21, P22, and P23), genome features such as GC content (49.0%), number of tRNAs (44), and number of pseudogenes (2 to 4) were similar among isolates and the previously sequenced *Xiphinematobacter* genomes (Table 1). Genome length ranged from 909,775 to 926,970 bp among isolates, and the number of predicted proteins ranged from 836 to 848 (Table 1). The average predicted gene length was ∼867 bp, which was somewhat smaller than average gene lengths in outgroup *Verrucomicrobia* (e.g., ∼1,015 to 1,041 bp); however, ortholog comparisons did not suggest an abundance of partial (i.e., recently inactivated) genes in *Xiphinematobacter* genomes.

**TABLE 1:** Genome features among *Xiphinematobacter* spp.

Sample name^*a*^	Genome length (bp)	GC content (%)	No. of genes	No. of proteins	No. of rRNAs	No. of tRNAs	No. of pseudogenes	NCBI accession no.
**P15**	917,845	49.0	893	844	3	46	3	CP068477
**P18**	915,441	49.0	896	847	3	46	4	CP068476
**P21**	926,970	49.0	899	848	3	48	2	CP068475
**P22**	909,775	49.0	885	836	3	46	3	CP068474
**P23**	917,278	49.0	888	839	3	46	2	CP068473
**P3-11** ^*b*^	734,636	49.0	794	752	3	39	30	CP068472
XipG	915,884	47.7	867	818	3	46	1	CP012665.1
Xip2	915,884	47.7	867	818	3	46	1	SRX1527792

a Names in bold denote isolates collected in this study.

b The genome for this sample was interpreted to be incomplete.

### Phylogenetic analysis of *Xiphinematobacter* isolates.

**FIG 1:**
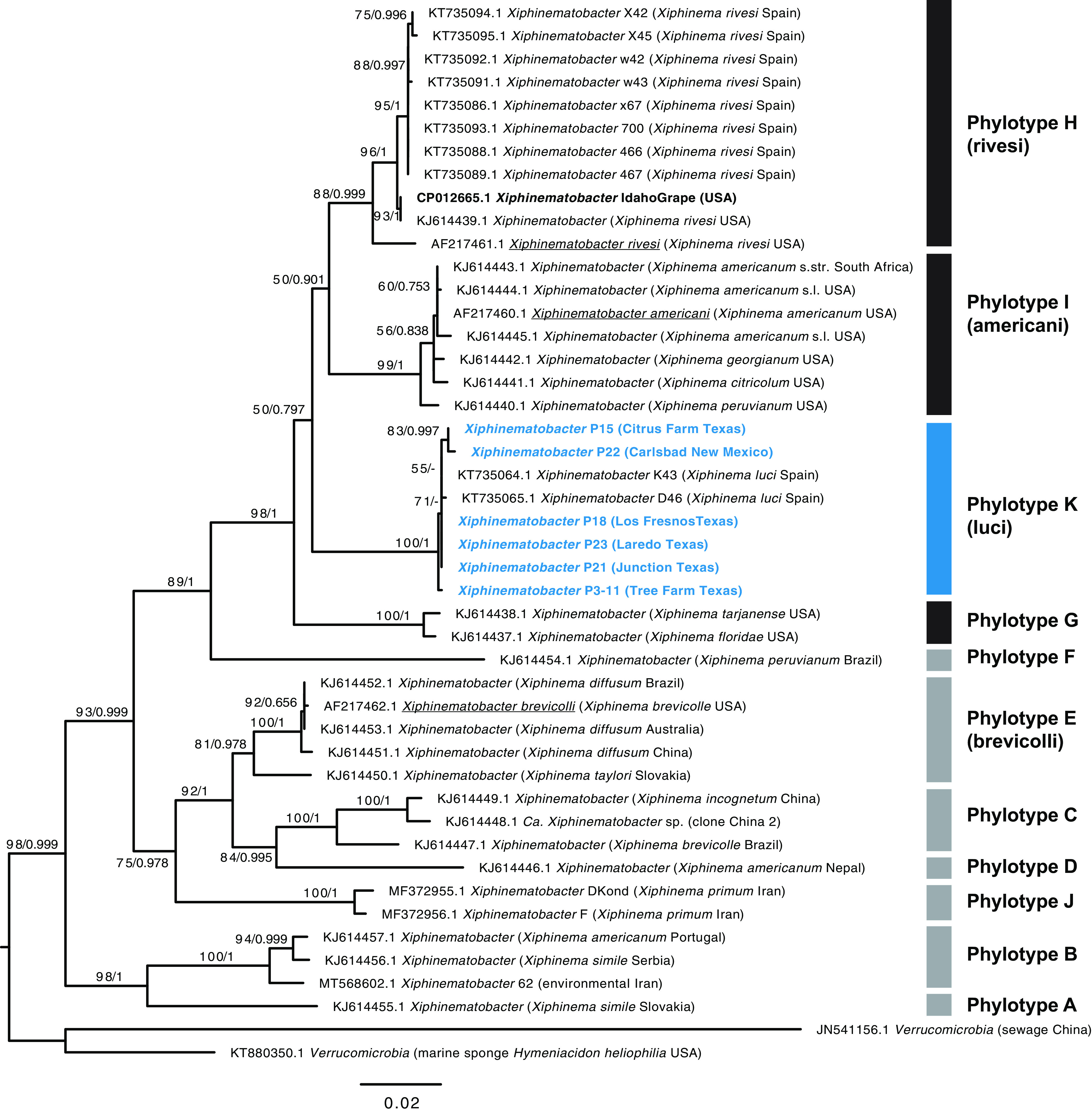
Phylogenetic tree of *Xiphinematobacter* spp. and verrucomicrobial outgroups, based on 1,513 aligned positions of the 16S rRNA gene, generated using RAxML GTR+G with 500 and MrBayes with GTR+G with 4 rate categories model, showing bootstrap replicate and posterior probability values on branches. Taxon names in blue bold font are from sequences obtained in this study. Phylotype designation based on reference 10 is shown alongside taxa, with dominant widespread North American groups indicated in black bars and other more globally diverse groups indicated with gray bars. Underlined taxon names indicate previously named *Xiphinematobacter* species. Black bold font indicates the isolate with previously sequenced genome.

Phylogenetic analysis of full-length 16S rRNA gene sequences for the 6 *Xiphinematobacter* isolates from this study and 38 other *Xiphinematobacter* sequences placed these isolates with high support into a single clade (100% bootstrap support and Bayesian posterior probability of 1), close to *Xiphinematobacter* isolates from *Xiphinema luci* collected in Spain (Fig. 1). This clade, designated “phylotype K,” is shown in the context of phylotypes proposed as *Xiphinematobacter* species by Lazarova et al. (10). Notably, the only *Xiphinematobacter* isolate whose genome had been sequenced previously, *Xiphinematobacter* sp. from Idaho Grape (GenBank accession number CP012665.1) falls into a distinct, strongly supported clade (phylotype H) that includes *Xiphinematobacter rivesi.* As shown in Fig. 1, the isolates from the current study and the Idaho Grape isolate clustered within a larger clade dominated by these symbionts from *Xiphinema* species that are widespread in North America (phylotypes G, H, I, and K), whereas other phylotypes occur in *Xiphinema* spp. that are more broadly distributed globally.

An analysis of a subsection of the 16S rRNA gene for which additional North American isolates have been sequenced (see Fig. S1 in the supplemental material) showed results consistent with full-length 16S rRNA analyses, supporting phylotype K. This clade included the *Xiphinematobacter* isolates from this study and the *Xiphinematobacter* isolates from *X. luci* collected in Spain as a sister clade to clade mt-C (11), which is notable as a distinct clade that is apparently not associated with nepoviruses.

10.1128/mSystems.01048-20.1 FIG S1 Maximum likelihood tree of *Xiphinematobacter* spp. and outgroups based on partial 16S rRNA sequences to incorporate broad isolates from North America, based on 519 aligned positions, using RAxML GTR+G with 500 bootstrap replicates with values of >50% shown on branches. Taxon names in blue bold font are from sequences obtained in this study. Phylotype designation based on reference 10 is shown alongside taxa, with dominant widespread North American groups indicated in black bars and other more globally diverse groups indicated with grey bars. Underlined taxon names indicate previously named *Xiphinematobacter* species. Black bold font indicates the isolate with previously sequenced genome. Download FIG S1, EPS file, 1.6 MB. Copyright © 2021 Myers et al. 2021 Myers et al. This content is distributed under the terms of the Creative Commons Attribution 4.0 International license.

Concatenated protein-coding gene-based phylogenetic analyses showed strong support for a close monophyletic group of *Xiphinematobacter* spp. with long branch distances to the closest outgroup *Verrucomicrobia* (see Fig. S2 in the supplemental material).

10.1128/mSystems.01048-20.2 FIG S2 Phylogenetic tree of *Xiphinematobacter* spp. and verrucomicrobial outgroups, based on 64,251 aligned nucleotide positions of 73 conserved protein-coding genes, generated using RAxML GTR+G with 500 and MrBayes with GTR+G with 4 rate categories model, showing bootstrap replicate and posterior probability values on branches. Taxon names in bold are from this study. Download FIG S2, EPS file, 0.7 MB. Copyright © 2021 Myers et al. 2021 Myers et al. This content is distributed under the terms of the Creative Commons Attribution 4.0 International license.

### Phylogenetic analysis and community profiles of *Xiphinema* nematodes.

**FIG 2:**
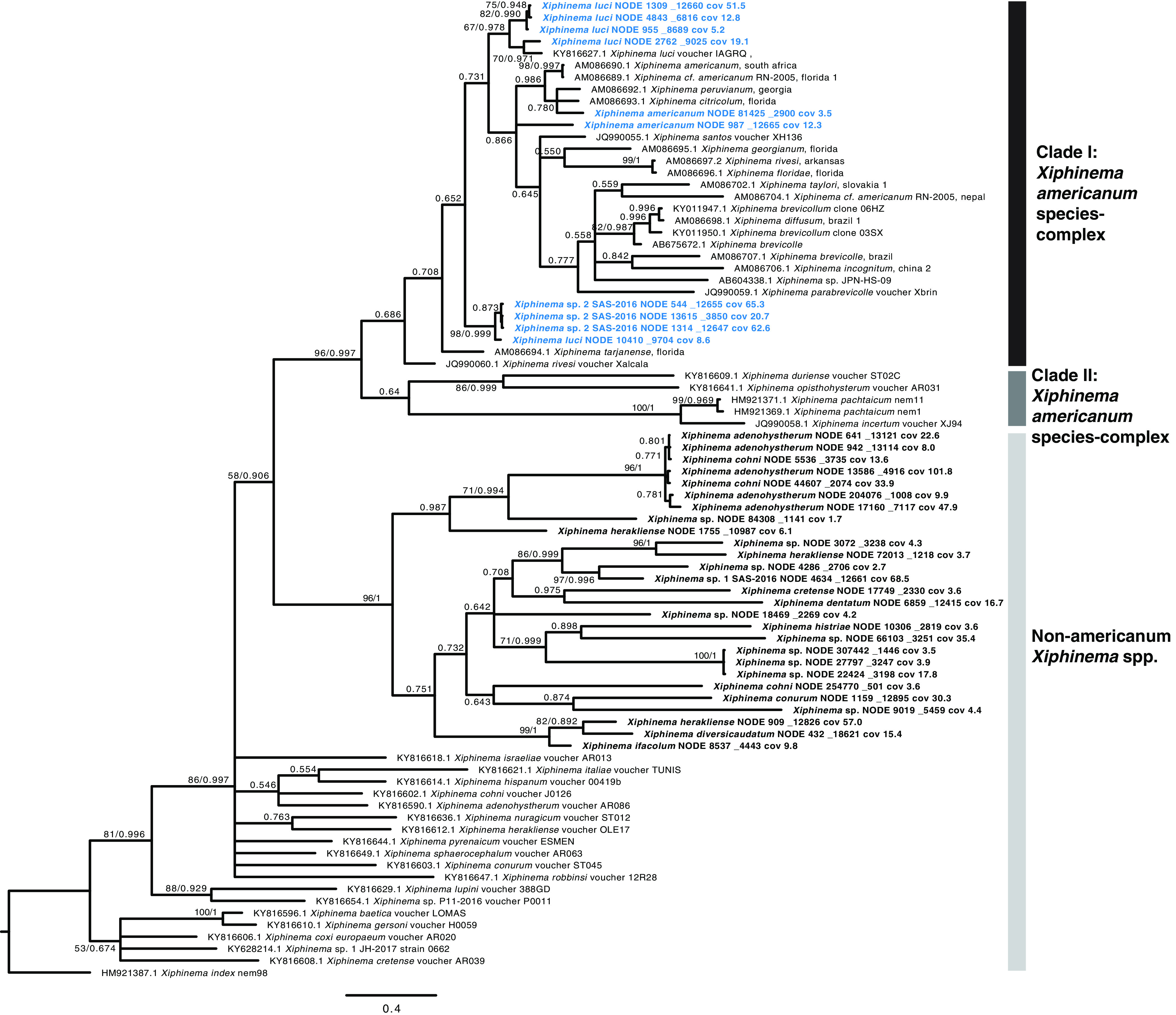
Phylogenetic tree of *Xiphinema americanum* species complex and non-*X. americanum Xiphinema* spp. showing the groups found in this study, based on 372 aligned positions of the partial cytochrome oxidase I (COI) gene, generated using Bayesian 50% majority rule in MrBayes with GTR+G with 4 rate categories model, and showing posterior probabilities and maximum likelihood bootstrap values from RAxML GTR+G with 500 and bootstrap replicates on the branches. Taxon names in bold are from sequences obtained in this study with *X. americanum* group representatives indicated in blue.

Phylogenetic analysis of the sequences with highest blastn similarity to the *Xiphinema* spp. COI gene from the 11 sampled locations produced a distinct pattern with 3 major clades (Fig. 2), representing previously characterized clades I and II of the *Xiphinema americanum sensu lato* species complex and a non-*X. americanum Xiphinema* spp. clade. Sequences from the current study fell into three subgroups within clade I *X. americanum* and one large and highly diverged group of non-*X. americanum Xiphinema* spp. (Fig. 2). The clade I *X. americanum* isolates were close matches to *X. luci*, *X. americanum*, and *Xiphinema* sp. 2 SAS-2016 from reference 45. There were no COI sequences from the current study with any similarity to clade II *X. americanum.*

**FIG 3:**
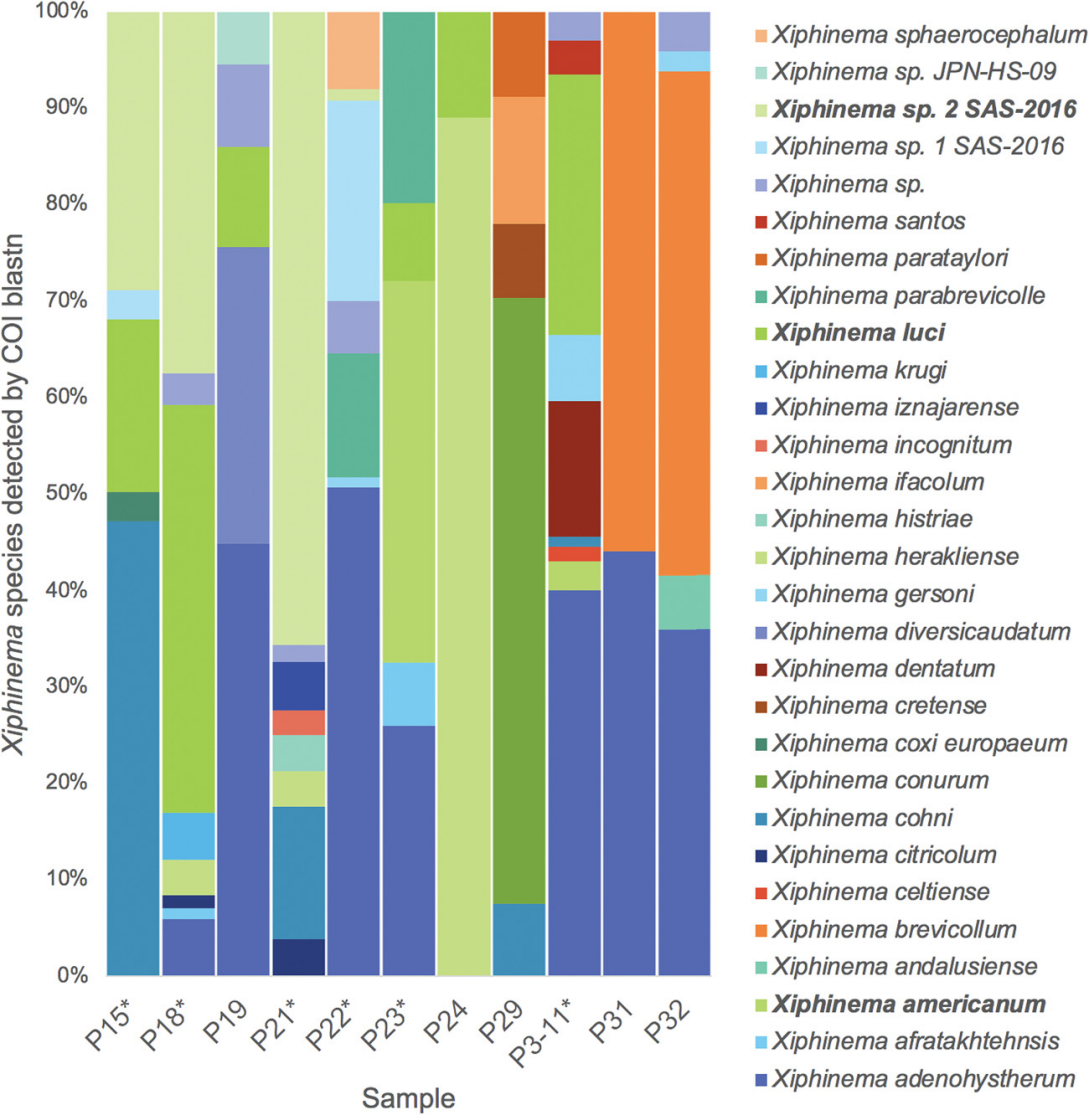
Relative percentage of each *Xiphinema* species in each sample, based on partial cytochrome oxidase I (COI) gene blast searches.

The relative proportions of COI matches in the 11 samples (considering sequencing coverage) are shown in Fig. 3 and indicate that most samples had 1 or 2 dominant *Xiphinema* species.

**FIG 4:**
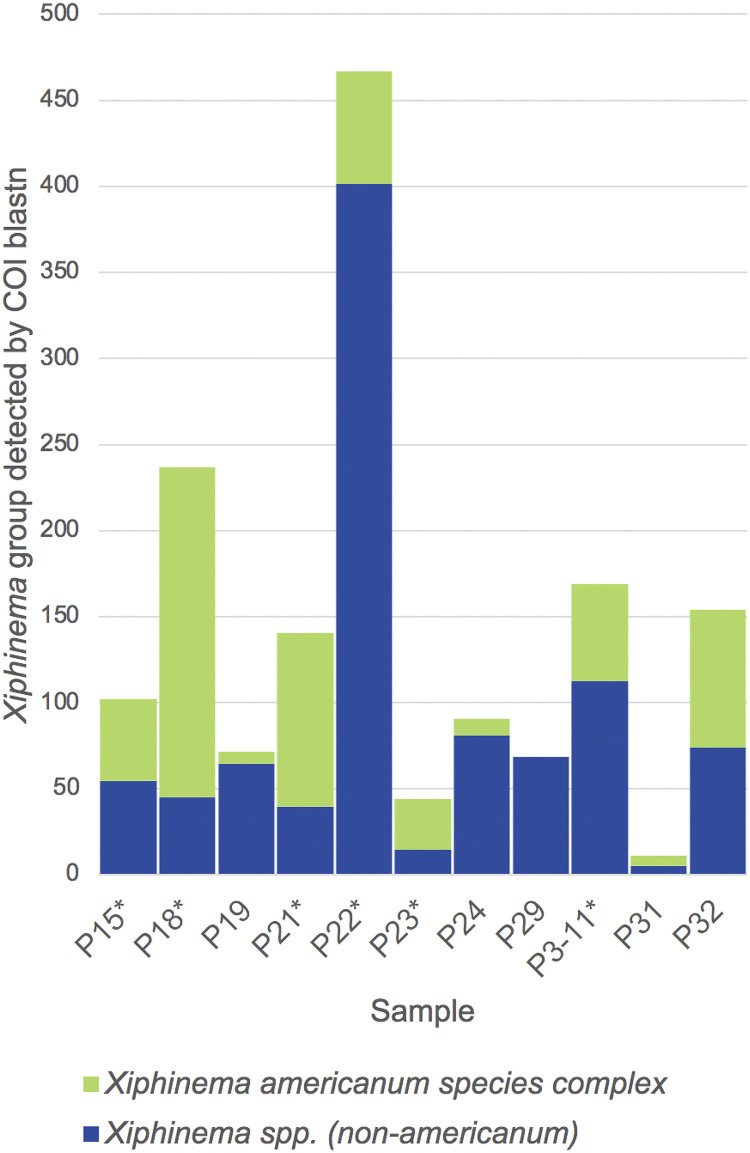
Total coverage of each *Xiphinema* type (*X. americanum* group versus non-*X. americanum* group) in each sample, based on partial cytochrome oxidase I (COI) gene blast searches.

Absolute coverage comparisons (Fig. 4) among samples indicate that *Xiphinematobacter*-positive samples (P15, P18, P21, P22, P23, and P3-11) generally had high coverage of *X. americanum* species-complex nematodes relative to *X. americanum Xiphinema* nematodes compared with *Xiphinematobacter*-negative samples (especially P19, P24, and P29) except for P32, which was dominated by the of *X. americanum* species complex nematode Xiphinema brevicollum, but did not carry matches close to *X. luci*.

### Genome repertoire and enrichment analysis of Xiphinematobacter isolates and outgroups.

Genome-wide sequence comparisons, including gene order and gene presence-absence comparisons, showed conserved synteny and similar gene compaction patterns and core genome repertoire among all eight *Xiphinematobacter* genomes, which differed distinctly from genomes of outgroup Verrucomicrobia (see Fig. S3 in the supplemental material).

10.1128/mSystems.01048-20.3 FIG S3 Complete gene presence and absence patterns for *Xiphinematobacter* spp. and outgroup *Verrucomicrobia* sp., generated using Roary and Phandango. Download FIG S3, EPS file, 0.8 MB. Copyright © 2021 Myers et al. 2021 Myers et al. This content is distributed under the terms of the Creative Commons Attribution 4.0 International license.

**FIG 5:**
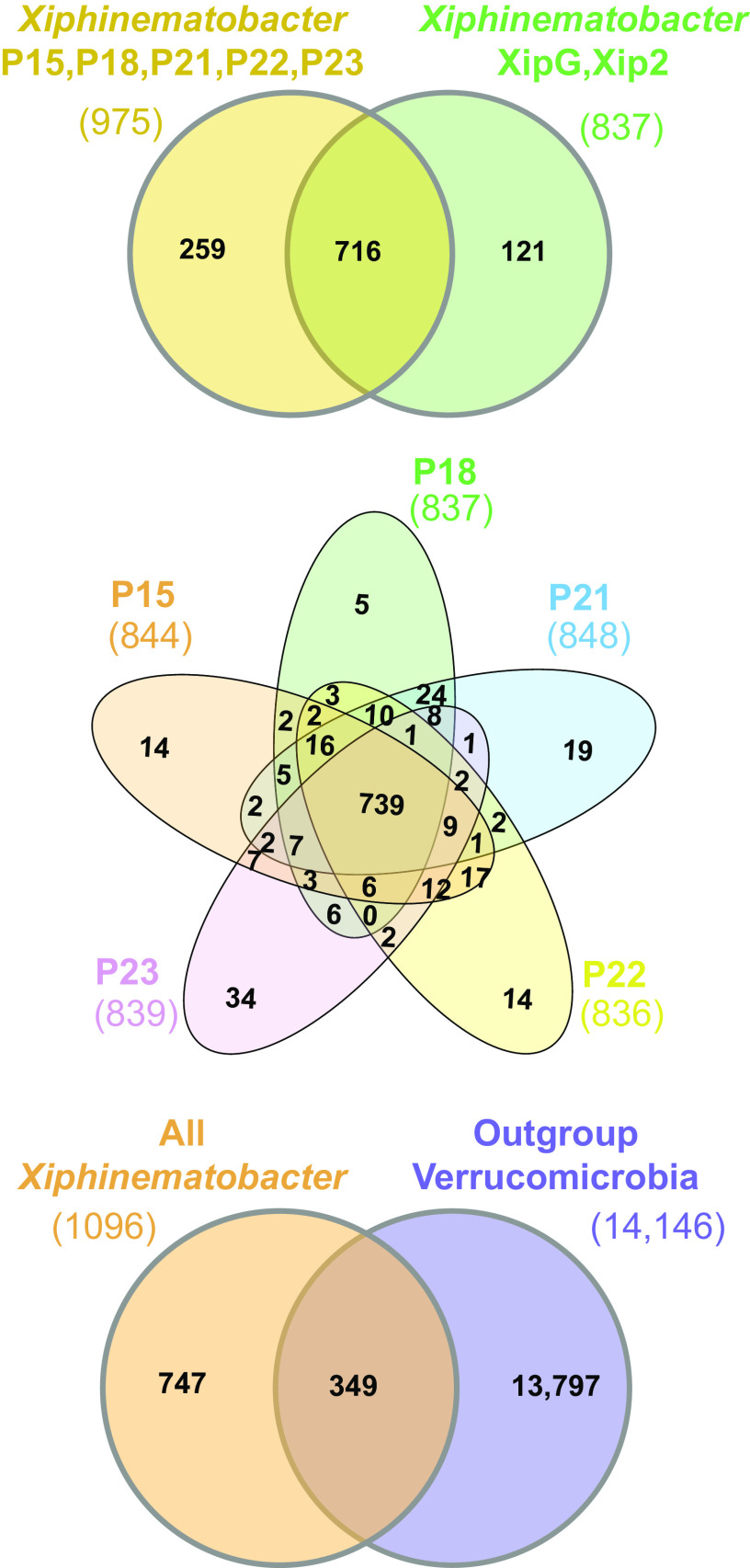
Pangenomes for *Xiphinematobacter* isolate from this study, previously sequenced *Xiphinematobacter*, and outgroup Verrucomicrobia, including *Spartobacteria bacterium* AMD-G4 (NEUK01000001.1), *Chthoniobacter flavus* Ellin428 (ABVL01000062.1), Terrimicrobium sacchariphilum NM-5 (NZ_BDCO01000003), and “*Candidatus* Udaeobacter sp. AEW3 Udaeo2_1” (JAALOD010000001.1).

Pangenome analyses (Fig. 5) revealed only 349 conserved genes out of 14,893 (2.3%) shared between *Xiphinematobacter* isolates and *Verrucomicrobia* outgroups. There were 747 genes unique to the *Xiphinematobacter* pangenome (i.e., not occurring in outgroups), of which a majority (470/747, 62.9%) were not annotated to function (i.e., predicted “hypothetical protein”). In contrast, the majority of genes (716 of 1,096, 65.3%) were conserved across the 7 fully sequenced *Xiphinematobacter* isolates, and among the 5 new genomes sequenced here, 75.8% (739 of 975) of genes were shared across isolates (Fig. 5). There were 379 core genes shared across all *Xiphinematobacter* species isolates but not present in outgroups, 254 genes unique to the pangenome of our 5 new *Xiphinematobacter* isolates, and 115 genes unique to the pangenome of the 2 previously sequenced *Xiphinematobacter* isolates (XipG and Xip2). However, from the 254 genes unique to the new isolates, only 10 of could be annotated with known gene function, while the others were annotated as hypothetical protein. Similarly, from the 115 genes unique to the previously sequenced isolates, only 5 genes could be annotated to a specific function.

**FIG 6:**
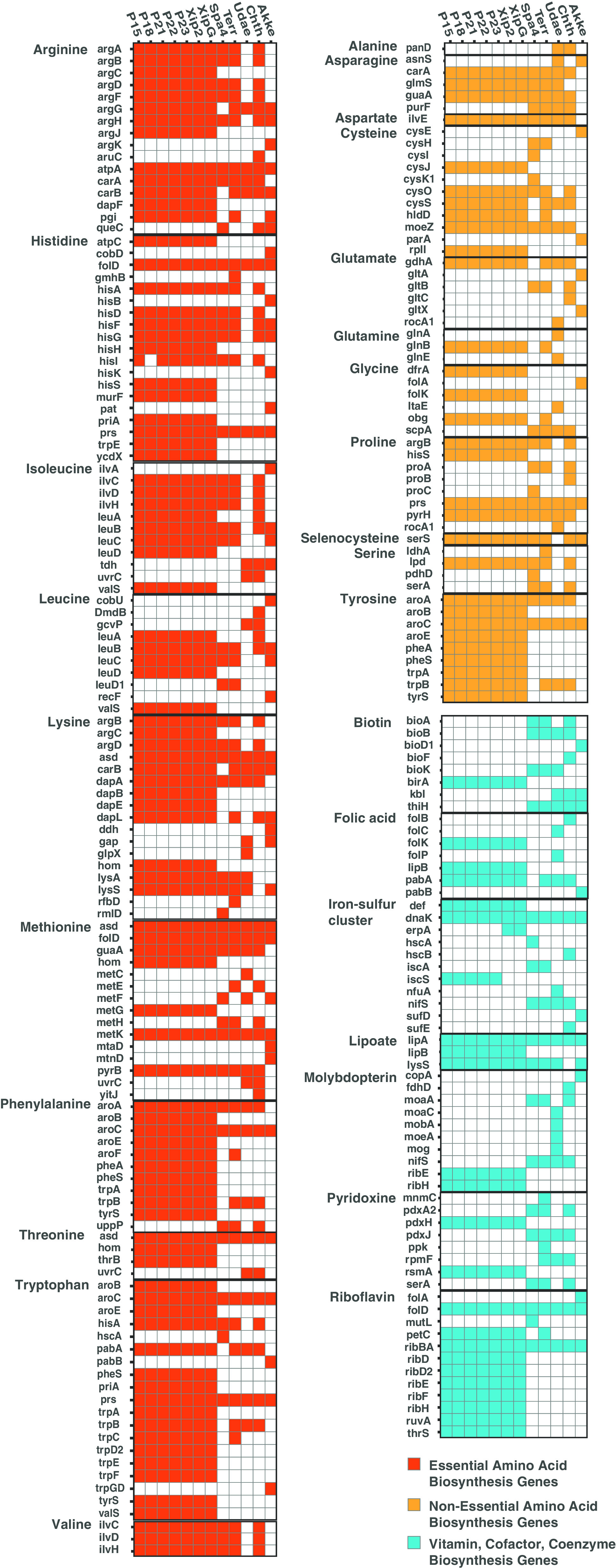
Amino acid and vitamin/cofactor biosynthesis gene presence and absence patterns for *Xiphinematobacter* isolates from this study (P15, P18, P21, P22, and P23), previously sequenced *Xiphinematobacter* spp. (XipG and Xip2), and outgroup *Verrucomicrobia* spp., including *Spartobacteria bacterium* AMD-G4 (NEUK01000001.1; Spa4), *Chthoniobacter flavus* Ellin428 (ABVL01000062.1; Chth), Terrimicrobium sacchariphilum NM-5 (NZ_BDCO01000003; Terr), “*Candidatus* Udaeobacter sp. AEW3 Udaeo2_1” (JAALOD010000001.1; Udae), and Akkermansia muciniphila YL44 (NZ_CP021420.1; Akke).

Specific gene presence-absence patterns for amino acid and vitamin/cofactor biosynthetic gene pathways among *Xiphinematobacter* isolates and *Verrucomicrobia* outgroups are depicted in Fig. 6. These patterns show a high level of conservation of genes for the 10 essential amino acid (EAA) biosynthesis pathways (arginine, histidine, isoleucine, leucine, lysine, methionine, phenylalanine, threonine, tryptophan, and valine), with lower conservation of genes in some of the nonessential amino acid (non-EAA) pathways and most of the vitamin/cofactor pathways. Among the essential amino acid pathway genes, methionine, histidine, and leucine appear to be missing several genes in *Xiphinematobacter* isolates, although the missing genes are conserved among the isolates. Among nonessential amino acids (alanine, asparagine, aspartate, cysteine, glutamate, glutamine, glycine, proline, selenocysteine, serine, and tyrosine), only genes for tyrosine appear to be well conserved; however, most of this pathway is shared with phenylalanine. Among the vitamins/cofactors, riboflavin and lipoate biosynthesis stand out as having largely intact genes in *Xiphinematobacter* sp. (Fig. 6).

From the 747 genes unique to the *Xiphinematobacter* pangenome (i.e., not occurring in outgroups) (Fig. 5), 262 protein-coding genes could be annotated beyond hypothetical protein. These genes were analyzed to assess functional enrichment compared with the background or “universe” genes for the combined *Xiphinematobacter* and *Verrucomicrobia* pangenome using topGO. The results (Table 2) showed significantly enriched GO terms (*P* values of <0.05) for 17 biological processes, 2 cellular compartments, and 6 metabolic functions. Most of these significantly enriched terms include housekeeping functions (e.g., cell division, regulation of cell shape, and cell cycle), but among these terms were also enriched an biosynthesis of several EAAs and vitamins, including aromatic amino acids, tryptophan, riboflavin, thiamine, and folic acid-containing compound biosynthesis.

**TABLE 2:** Significantly enriched gene ontology categories among *Xiphinematobacter* species^*a*^

GO_iD by category	Term	No. of annotated genes	No. of significant genes	No. of genes expected	*P* value
Biological processes					
GO:0051301	Cell division	33	14	3.95	7.8e-06
GO:0008360	Regulation of cell shape	36	12	4.31	0.00025
GO:0007049	Cell cycle	33	12	3.95	0.00043
GO:0015937	Coenzyme A biosynthetic process	5	4	0.6	0.00064
GO:0009073	Aromatic amino acid family biosynthetic process	30	12	3.59	0.00213
GO:0009423	Chorismate biosynthetic process	10	5	1.2	0.00237
GO:0009252	Peptidoglycan biosynthetic process	34	10	4.07	0.00243
GO:0015986	ATP synthesis coupled proton transport	7	4	0.84	0.00372
GO:0009231	Riboflavin biosynthetic process	9	4	1.08	0.01123
GO:0009396	Folic acid-containing compound biosynthetic process	10	4	1.2	0.01177
GO:0009245	Lipid A biosynthetic process	17	5	2.04	0.03069
GO:0000162	Tryptophan biosynthetic process	7	3	0.84	0.03230
GO:0043165	Gram-negative-bacterium-type cell outer membrane assembly	7	3	0.84	0.03230
GO:0008299	Isoprenoid biosynthetic process	16	4	1.92	0.03294
GO:0009228	Thiamine biosynthetic process	13	4	1.56	0.04488
GO:0006364	rRNA processing	22	7	2.63	0.04615
GO:0043093	Ftsz-dependent cytokinesis	8	3	0.96	0.04759
Cellular components					
GO:0016021	Integral component of membrane	441	61	51.26	0.00095
GO:0032153	Cell division site	7	3	0.81	0.03042
Metabolic functions					
GO:0003723	RNA binding	121	27	13.61	0.0020
GO:0046933	Proton-transporting ATP synthase activity, rotational mechanism	7	4	0.79	0.0031
GO:0005524	ATP binding	362	50	40.71	0.0145
GO:0003887	DNA-directed DNA polymerase activity	8	3	0.9	0.0418
GO:0000049	tRNA binding	26	6	2.92	0.0457
GO:0016757	Transferase activity, transferring glycosyl groups	85	17	9.56	0.0491

a Based on topGO gene ontology enrichment analysis among *Xiphinematobacter* species, based on genes unique to the *Xiphinematobacter* pangenome that do not occur in outgroups.

### Polymorphism analysis within and between *Xiphinematobacter* isolates.

**FIG 7:**
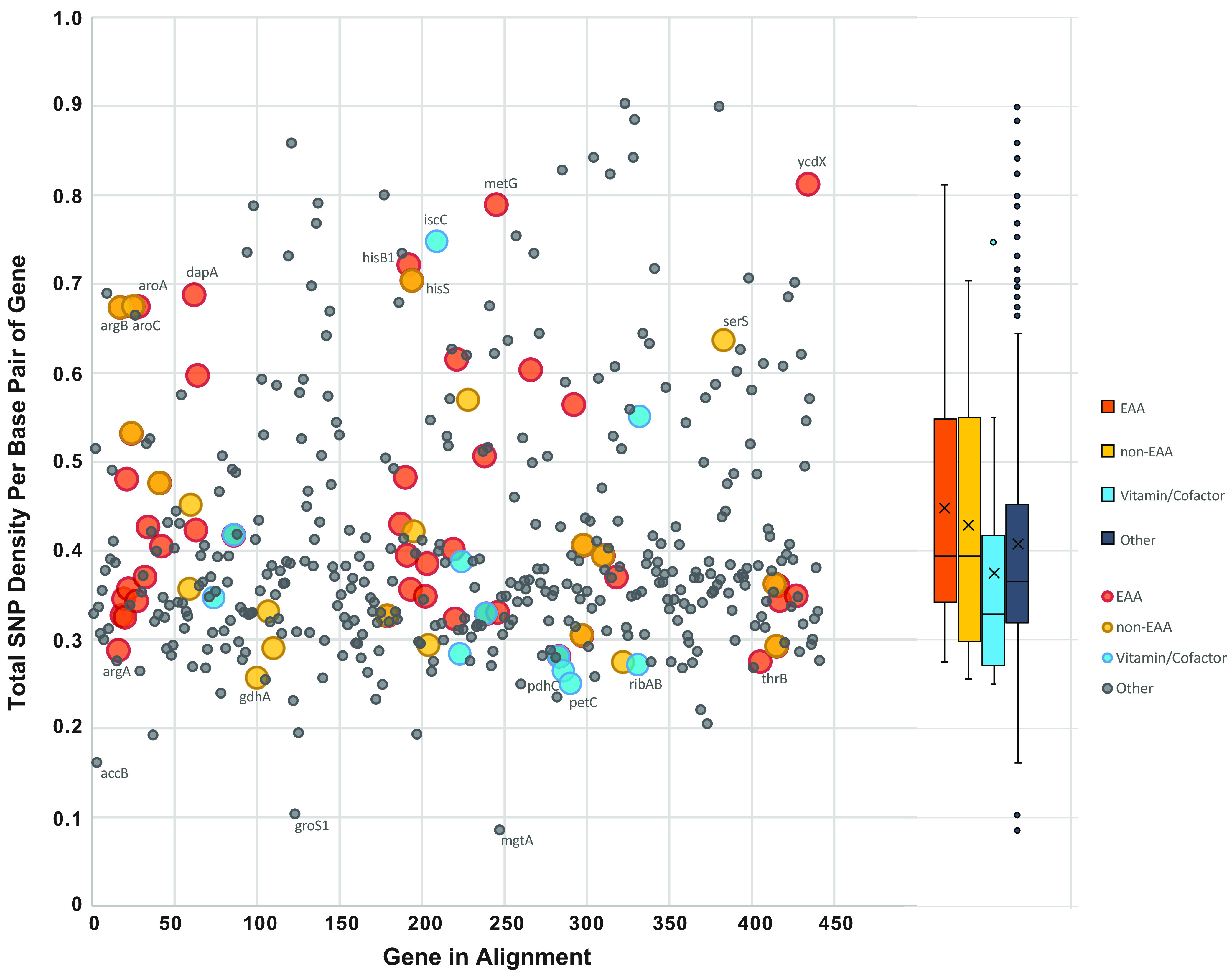
Single nucleotide polymorphism (SNP) density across conserved genes from eight *Xiphinematobacter* genomes compared in this study, across 441 genes, showing amino acid and vitamin/cofactor biosynthesis gene SNP density patterns. Mann-Whitney U test showed significant difference between essential amino acid (EAA) and vitamin/cofactor groups and between EAA and background (other) genes. Boxplot (right) shows 1st to 3rd quartile range, median (line), mean (x), and outliers.

Single nucleotide polymorphism (SNP) density analysis across 441 conserved genes from 8 *Xiphinematobacter* genomes (Fig. 7), calculated using snp-sites software, showed several genes with exceptionally high SNP density, including some amino acid and vitamin/cofactor biosynthesis genes. SNP density was slightly higher for amino acid biosynthesis genes than for vitamin/cofactor genes and background genes (“other”) not falling within these categories; Mann-Whitney U test of these categorized SNP densities showed significant differences, including EAA versus vitamin/cofactor (Z-score, 2.03461; *P* = 0.02118) and EAA versus other (Z-score, −2.06105; *P* = 0.0197). Notably high SNP density genes included the EAA genes *aroA*, *aroC*, *argB*, *dapA*, *hisB1*, *hisS*, *metG*, and *ycdX*; the non-EAAs *aroC*, *hisS*, and *serS*; and the vitamin gene *iscC*. Notably low SNP density genes included *argA*, *gdhA*, *pdhC*, *petC*, *ribAB*, and *thrB*.

### Gene ontology enrichment analysis of phenotype-associated loci from de Bruijn GWAS.

Using DBGWAS for 7 *Xiphinematobacter* genomes, 51,449 unique SNPs were detected in de Bruijn graphs, from which 4,193 SNPs (de Bruijn kmers) showed a statistically significant association with the host *Xiphinema* group (specifically, *X. rivesi* or *X. luci*) (see Table S3 in the supplemental material). Functional enrichment analysis was performed on the set of 3,440 of these SNPs for which the gene could be annotated (i.e., removing hypothetical proteins) and a gene ontology (GO) category could be assigned. GO enrichment tests were performed on the 525 genes containing these SNPs by comparing the associated gene set to the background or universe genes for *Xiphinematobacter* and *Verrucomicrobia* sp. using topGO. The results (Table 3) showed 25 significantly enriched GO biological processes (*P* < 0.05), which included several housekeeping functions (e.g., translation, *de novo* UMP biosynthesis, and cell division). Among the 25 significantly enriched processes, 7 were for EAA (histidine, arginine, isoleucine, valine, threonine, leucine, and tryptophan) biosynthesis, 1 was aromatic amino acid biosynthesis (tyrosine, phenylalanine, and tryptophan), and 1 was for biosynthesis of the vitamin thiamine.

**TABLE 3:** Significantly enriched gene ontology categories from DBGWAS analysis^*a*^

GO_iD	Term	No. of annotated genes	No. of signficant genes	No. of genes expected	*P* value
GO:0006412	Translation	100	71	23.89	8.0e-19
GO:0000105	Histidine biosynthetic process	10	9	2.39	1.0e-05
GO:0044205	“*De novo*” UMP biosynthetic process	7	7	1.67	2.7e-05
GO:0051301	Cell division	33	17	7.88	6.8e-05
GO:0006526	Arginine biosynthetic process	11	9	2.63	0.00017
GO:0015937	Coenzyme A biosynthetic process	5	5	1.19	0.00055
GO:0008360	Regulation of cell shape	36	17	8.6	0.00076
GO:0007049	Cell cycle	33	14	7.88	0.00079
GO:0009073	Aromatic amino acid family biosynthetic process	30	22	7.17	0.00144
GO:0009423	Chorismate biosynthetic process	10	7	2.39	0.00169
GO:0042254	Ribosome biogenesis	36	14	8.6	0.00239
GO:0015986	ATP synthesis coupled proton transport	7	6	1.67	0.00265
GO:0009097	Isoleucine biosynthetic process	11	7	2.63	0.00375
GO:0009245	Lipid A biosynthetic process	17	9	4.06	0.00548
GO:0009252	Peptidoglycan biosynthetic process	34	14	8.12	0.01007
GO:0019288	Isopentenyl diphosphate biosynthetic process, methylerythritol 4-phosphate pathway	5	4	1.19	0.01012
GO:0009099	Valine biosynthetic process	5	4	1.19	0.01012
GO:0009088	Threonine biosynthetic process	5	4	1.19	0.01012
GO:0006351	Transcription, DNA-templated	148	18	35.36	0.01196
GO:0006096	Glycolytic process	18	9	4.3	0.02044
GO:0009098	Leucine biosynthetic process	9	5	2.15	0.03067
GO:0006396	RNA processing	63	25	15.05	0.04495
GO:0000162	Tryptophan biosynthetic process	7	4	1.67	0.04821
GO:0009228	Thiamine biosynthetic process	13	6	3.11	0.04879
GO:0006353	DNA-templated transcription, termination	6	5	1.43	0.04929

a Based on topGO gene ontology enrichment analysis for *Xiphinematobacter*, based on genes that are significantly associated with host phenotype (*Xiphinema* species clade) in de Bruijn genome wide association (DBGWAS) SNP analysis.

10.1128/mSystems.01048-20.7 TABLE S3 Table of kmers containing alleles that are significantly associated with different host phenotype (*Xiphinema* species) based on de Bruijn genome-wide association (DBGWAS) analysis. Download Table S3, XLSX file, 1.1 MB. Copyright © 2021 Myers et al. 2021 Myers et al. This content is distributed under the terms of the Creative Commons Attribution 4.0 International license.

### Polymorphism density for associated loci from de Bruijn GWAS.

**FIG 8:**
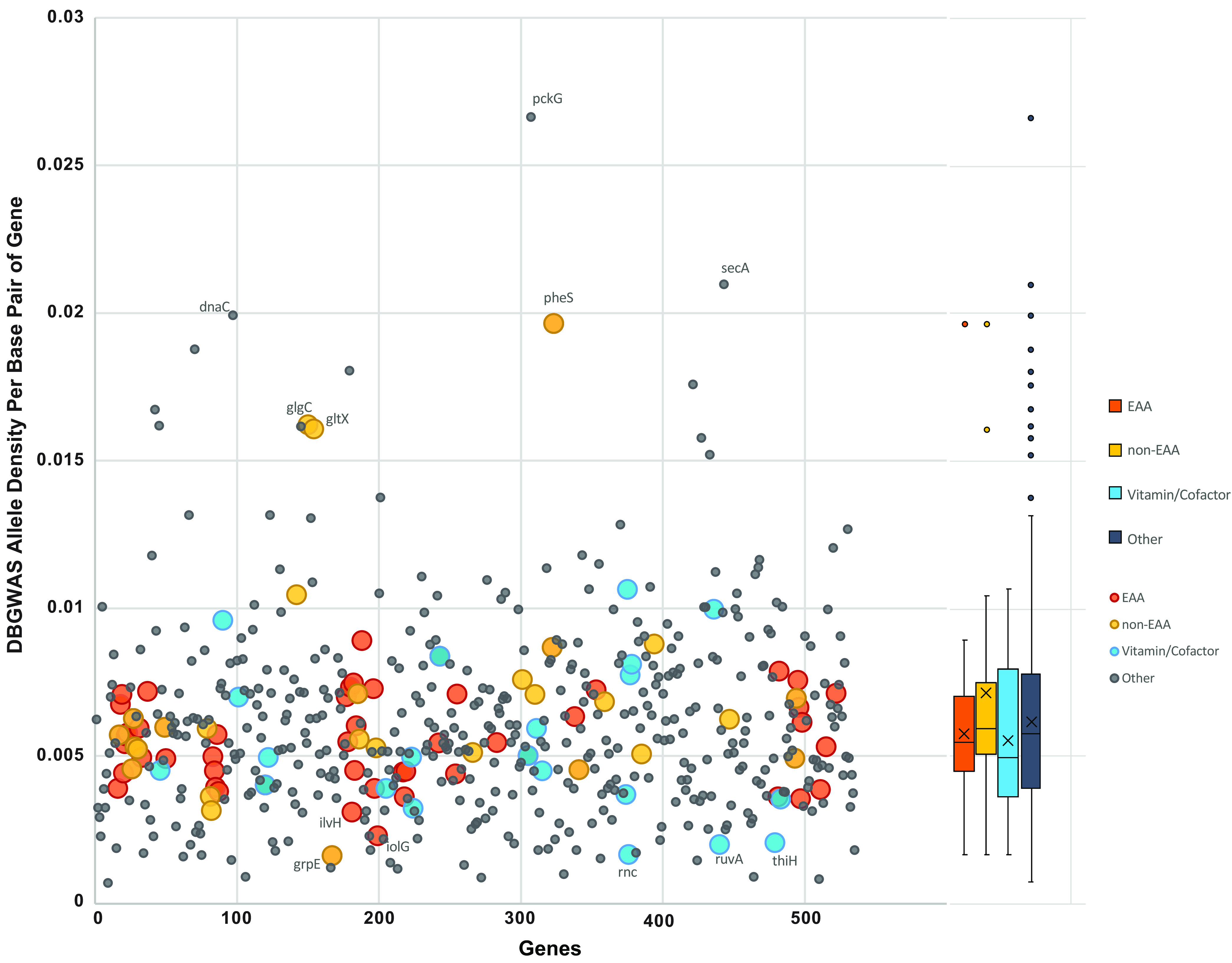
Density of de Bruijn genome-wide association (DBGWAS) SNPs that are significantly associated with different host phenotype (*Xiphinema* species clade) plotted for 536 genes, showing amino acid and vitamin/cofactor biosynthesis gene SNP density patterns. Mann-Whitney U test showed significant differences between non-EAA and vitamin/cofactor genes. Boxplot (right) shows 1st to 3rd quartile range, median (line), mean (x), and outliers.

Our higher-resolution analysis of these data showed the density of the significantly associated DBGWAS SNPs per gene (Fig. 8). These host-phenotype-associated SNP sites showed a trend in contrast to the total SNP density (Fig. 7), with essential amino acid gene DBGWAS SNPs having lower density, although this was not significant and this density matched that of the background genes (Fig. 8). Mann-Whitney U test of these categorized DBGWAS SNP densities showed a significant difference between non-EAA versus vitamin/cofactor (Z-score, 1.66675; *P* = 0.04746). Notably high DBGWAS SNP density genes included the non-EAA genes *pheS*, *glgC*, and *gltX*. Notably low-SNP genes include the vitamin genes *rnc*, *ruvA*, and *thiH*; and amino acid genes *grpE*, *ilvH*, and *iolG*.

### Analysis of signatures of selection across gene groups.

**FIG 9:**
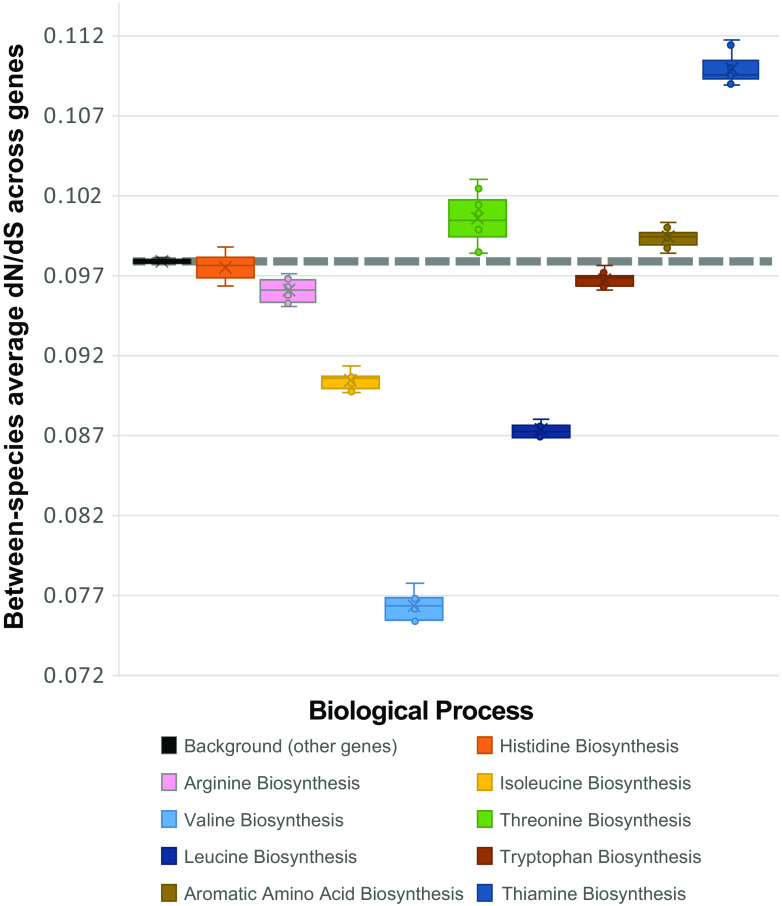
Between-species dN/dS in *Xiphinematobacter* sp. for select amino acid and vitamin/cofactor biosynthesis pathways, identified as enriched from de Bruijn genome-wide association (DBGWAS SNP) gene ontology analysis, compared to remainder of the genome (i.e., background), and calculated using the Nei and Gojobori 1986 method for dN/dS in codeML.

The ratio of nonsynonymous and synonymous substitutions (dN/dS) between *Xiphinematobacter* clades for genes categorized into individual “nutritional” biosynthesis pathways identified in Table 2 are shown in Fig. 9. Compared with typical background genome-wide values, dN/dS was lower in genes for arginine, isoleucine, leucine, valine, and tryptophan biosynthesis. In contrast, dN/dS was higher than background levels in genes for threonine and thiamine biosynthesis and aromatic amino acids.

Within species of *Xiphinematobacter*, isoleucine and valine again showed lower dN/dS than background, but arginine showed a higher dN/dS, while other trends in Fig. 9 were not observed, with most genes having values close to that of the background (see Fig. S4 in the supplemental material).

10.1128/mSystems.01048-20.4 FIG S4 Within-species dN/dS for *Xiphinematobacter* species for amino acid and vitamin/cofactor biosynthesis pathways and remaining genes (i.e., background), calculated using the Nei and Gojobori 1986 method for dN/dS in codeML. Download FIG S4, EPS file, 0.6 MB. Copyright © 2021 Myers et al. 2021 Myers et al. This content is distributed under the terms of the Creative Commons Attribution 4.0 International license.

### Analysis of site-specific positive selection across gene groups.

**FIG 10:**
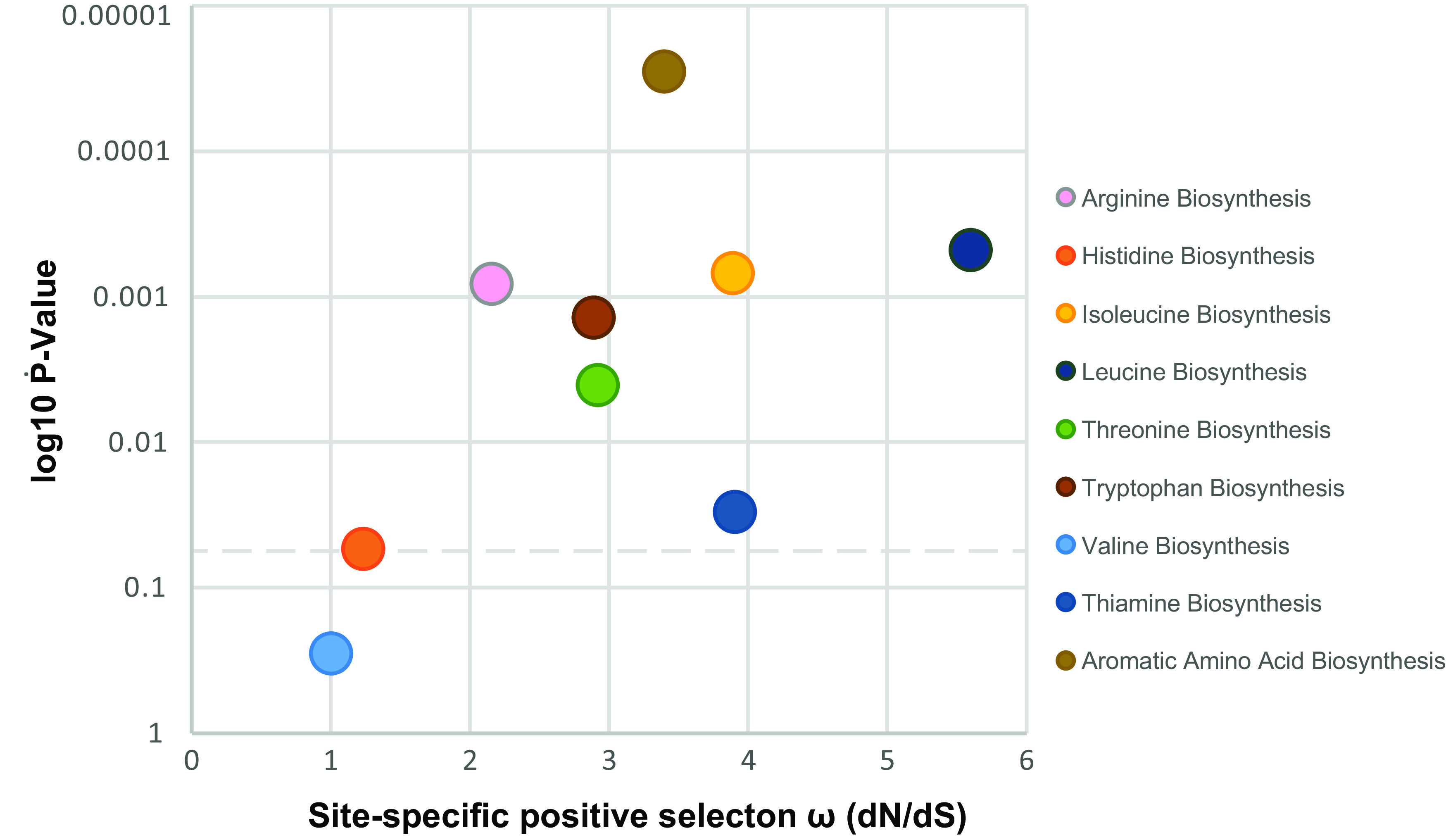
Strength of positive selection and positively selected sites on amino acid and vitamin/cofactor biosynthesis pathway genes among *Xiphinematobacter* species based on the likelihood ratio test (LRT) for nested models of positive selection (M7 = beta -; 10 categories versus M8 beta ω > 1; 11 categories) calculated in codeML. Dotted line indicates a *P* value of 0.05.

Site-specific tests of positive selection in the same groups of genes were calculated in codeML using the likelihood ratio test (LRT) for nested models of positive selection (M7 = beta −; 10 categories versus M8 beta ω of >1; 11 categories), with results shown in Table S4 in the supplemental material, and Fig. 10 *P* values showed significance at <0.05 for ω of >1 (i.e., positively selected sites) for all biosynthesis pathways except valine biosynthesis. Histidine was the second lowest in ω and second highest in *P* value, whereas the most outstanding high ω pathways were, in descending order, leucine, thiamine, isoleucine, aromatic amino acids, threonine, and tryptophan.

10.1128/mSystems.01048-20.8 TABLE S4 Table of positively selected sites from the LRT test in codeML. Download Table S4, XLSX file, 0.01 MB. Copyright © 2021 Myers et al. 2021 Myers et al. This content is distributed under the terms of the Creative Commons Attribution 4.0 International license.

## DISCUSSION

A wide range of endosymbionts play critical roles in their hosts (2, 4, 46). Familiar examples include *Buchnera* sp. (*Enterobacteriaceae*) which supplement missing essential amino acids in their aphid hosts and *Wolbachia* (*Alphaproteobacteria*) which are either reproductive parasites or mutualists in insects and nematodes. However, for some unculturable obligate endosymbionts living within nonmodel hosts, such as *Xiphinematobacter* sp., ecological and physiological experiments are extremely challenging; therefore, studies of the endosymbiont role depend critically on genomics. Here, we present novel insights into the role of *Xiphinematobacter* sp. by focusing on genomics-based approaches to understand the selective pressure on nutritional supplementation pathways.

Based on our comparative genomic analyses of genome repertoire and enrichment, a major pattern that emerged was the conservation of several essential nutrient biosynthetic pathway genes across *Xiphinematobacter* isolates compared with the outgroup *Verrucomicrobia*, and we also found evidence of directional selection in these pathways using population genomics. Gene repertoire data showed these highly reduced *Xiphinematobacter* endosymbiont genomes have universally conserved biosynthesis pathways for most essential amino acids, tyrosine, and several vitamins/cofactors, with statistically overrepresented GO terms for tryptophan, aromatic amino acid, riboflavin, folate, and thiamine biosynthesis, despite an endosymbiosis-driven pattern of gene loss, expanding previous results based on a single *Xiphinematobacter* genome (8). While endosymbionts often evolve reduced genome repertoires in the absence of purifying selection on genes for factors supplied by the host (5, 6), it is exceptional for the metabolically expensive essential amino acid and vitamin synthesis pathways to be conserved unless the symbiont is provisioning these nutrients to supplement its host diet.

Many examples of highly conserved pathways for *de novo* biosynthesis of essential amino acids and vitamins/cofactors in endosymbionts occur in insects feeding strictly on low amino acid or low vitamin diets, such as phloem (47) or blood, in which the symbionts match the host nutritional demands (48) and facilitate host plant use (1). Our findings of a disproportionate conservation of these pathways hint at a similar role of *Xiphinematobacter* sp. in supplementing these essential nutrients in its nematode diet. These results are supported by the gut wall tissue distribution of this symbiont (8, 49). While nematodes, like other animals, are unable to synthesize these essential molecules *de novo*, nutritional mutualism in plant-feeding nematodes seems to be rare. This is likely because most plant-feeding and plant-parasitic nematodes have sophisticated adaptations to secrete plant cell wall-digesting enzymes that release plant cell contents, which are rich in nutrients (50), explaining the observation that most *Xiphinema* species do not appear to require symbiotic microbes (51–53). Yet, based on strong coevolutionary patterns and 100% prevalence globally in individuals and nominal species in the *X. americanum* complex clade I and most of clade II (9, 11, 34, 35), *Xiphinematobacter* sp. appears to be a long-standing resident mutualist (7, 8). Details of *Xiphinema* feeding patterns suggest some species may specialize on drinking from vascular bundle and phloem (54), potentially explaining nutrient deficiencies that would demand supplementation by a symbiont.

Polymorphism analyses among isolates of *Xiphinematobacter* sp., including gene- and pathway-based SNP density, showed significantly increased standing genetic variation and allele fixation in EAAs compared with vitamin/cofactor and background genes, suggesting a potentially enhanced adaptive potential in EAA biosynthesis pathways. Specific genes with higher SNP density included three genes for histidine (none known to be rate limiting), two critical genes for phenylalanine and tryptophan (*aroA* and *aroC*; encoding 3-phosphoshikimate 1-carboxyvinyltransferase and chorismite synthase, respectively), two for lysine (*argB* encoding acetylglutamate kinase, and *dapA* encoding the lysine biosynthesis rate-limiting product 4-hydroxy-tetrahydrodipicolinate synthase) (55), and one for methionine (*metG* encoding methionyl-tRNA synthase that may affect methionine biosynthesis through altering exogenous methionine levels which generally repress *met* synthesis genes; however, such feedback inhibition likely varies between species and is still poorly understood) (56). The less critical genes in these pathways may accumulate SNPs due to relative relaxation of purifying selection; however, it is unclear why the critical or rate-limiting genes should accumulate more SNPs, unless these mutations accumulate through positive selection. Lower SNP-density genes included two for riboflavin, including the critical riboflavin biosynthesis protein RibAB, which is consistent with riboflavin synthesis being critical, as has been proposed for other symbionts (57).

However, the findings above include both synonymous and nonsynonymous sites, whereas gene ontology (GO) enrichment analyses supported this pattern and focused on the set of variants that were fixed between groups. For example, GO analysis of de Bruijn-based genome-wide association (GWAS) SNPs showed 7 out of 25 (32%) significant terms were for essential amino acid biosynthesis (histidine, arginine, isoleucine, valine, threonine, leucine, and tryptophan). Because this association analysis for DBGWAS SNPs focused on those significantly associated with host species clade as the phenotype (i.e., *X. rivesi* clade versus *X. luci* clade), the focus emphasized fixed SNPs in these symbionts within the *X. americanum* species complex. Thus, the EAA biosynthesis overrepresentation in DBGWAS SNPs in *Xiphinematobacter* sp. is indicative of positive selection driving the fixation of alleles for these functions. These findings add to a growing list of studies demonstrating adaptation through population genomics of host-associated microbes (38, 40, 41, 43, 58–60). Densities of DGBWAS SNPs suggested that fixation in non-EAA biosynthetic pathways was greater, overall, than fixation in vitamin/cofactor pathways, perhaps due to a lower evolutionary rate or higher purifying selection in the latter. Together, overrepresentation of DBGWAS SNPs in the biosynthesis of EAAs and several vitamin/cofactor processes suggests more rapidly evolving fixation at the species level—a finding consistent with higher overall SNP density, high retention of these genes in gene presence/absence data, and selection, which is discussed below.

Selection tests (e.g., dN/dS, LRT test) showed differential signatures of positive and purifying selection both across biosynthetic pathways and at specifically selected sites. For example, some pathways appeared to be under lower selective constraints (notably thiamine, threonine, and aromatic amino acid biosynthesis) than background dN/dS, while other pathways appeared to be under higher constraint (i.e., purifying selection), such as valine, leucine, and isoleucine, and to a lesser extent, arginine and tryptophan. This finding suggests there are dynamic evolutionary effects on these conserved nutritional pathways—noting that background genome dN/dS was substantially lower than 1, indicating the *Xiphinematobacter* symbiont genome is under strong purifying selection. Mirroring this result, within-species dN/dS was also low for valine and isoleucine, suggesting more constrained evolution than background on these biosynthetic pathways and implying their potential importance across *Xiphinematobacter* sp. In comparative LRT tests of site-specific positive selection, assuming selection (ω) is constant across branches in the phylogeny of *Xiphinematobacter* isolates, we found strong statistical support for positive selection in numerous sites in all the pathways above, except for valine, which is consistent with valine’s overall stronger purifying selection. Together, these results suggest higher positive selection, ω, in pathways for the biosynthesis of leucine and isoleucine, with stronger purifying selection between species, but high site-specific positive selection, in contrast to biosynthesis pathways for thiamine and aromatic amino acids, which showed overall higher dN/dS as well as high (i.e., ω > 1) site-specific positive selection, emphasizing likely directional selection on these fixed divergences in these pathways. Between-isolate positive selection in this case may indicate that different hosts may place different selection pressures on these genes (e.g., due to hosts feeding on different plants or living in different habitats). Such analyses of selection on candidate microbial mutualism functions are rare (43, 61) but can be powerful when combined with manipulative experiments (60) and studies of molecular regulation between host and mutualist (62–64) and could form targets for exploration of new methods to control these pests.

The phylogenetic results were consistent with those of previous studies suggesting *Xiphinematobacter* clades codiversify with their hosts (9–11, 34, 35, 45) due to high fidelity vertical transmission of this obligate mutualist. Furthermore, phylogenies showed our new *Xiphinematobacter* isolates formed a well-supported monophyletic clade in all analyses, supporting the distinct species of *Xiphinematobacter*. These isolates had similar 16S rRNA sequences (<0.7% divergence) and similar genomic features (similar gene content, high synteny, and identical GC content). Comparing 16S rRNA divergences between close relatives to this clade (e.g., 4.0% to *Xiphinematobacter rivesi*, 4.1% to *Xiphinematobacter* from Idaho Grape, and 4.6% to *Xiphinematobacter americani*) and using fossil-based calibration from a *Wolbachia* sp. from apid bees (65) that estimates 216 million years divergence per ∼2.8% 16S rRNA divergence, we estimate this clade may be ∼308 million years old, perhaps diverging in the Carboniferous Period. A fascinating aspect of the *Xiphinema-Xiphinematobacter* symbiosis is the possibility of a replacement symbiont from among the *Burkholderiaceae* which was discovered in Xiphinema pachtaicum, Xiphinema incertum, Xiphinema astaregiense, Xiphinema parapachydermum, Xiphinema vallense, and Xiphinema madeirense in Spain and Iran (9, 34) but is not yet found in North America. However, we did not find reads or contigs with blastn similarity to *Burkholderia*-like symbionts; although, our study did not recover any matches to this symbiont.

To date, there have been few field surveys of nematodes and their microbes (66, 67), but the success of *Xiphinematobacter* and *Xiphinema* spp. recovery from field sites in this study demonstrates the effectiveness of community genome skimming for survey purposes while simultaneously yielding quality data for deeper analyses, such as phylogenomics and comparative and population genomics. Our census result showing *Xiphinematobacter* sp. in half of our sampled sites is consistent with this endosymbiont being common in soils (29, 30). Our results emphasize an advantage of genome skimming for surveys compared with PCR for long-evolved soil-dwelling endosymbionts which may have priming site differences and inhibitors from soil contaminants (9, 68–70). Although the skimming method does not directly assess host-symbiont combinations, they may be analyzed in complementary studies, for example using Hi-C or other technologies (71–73).

In conclusion, this study presents novel insight into the *Xiphinema-Xiphinematobacter* symbiosis, which closely mirrors nutrient provisioning symbioses in hemipteran insects and highlights convergent roles in the evolution of microbiota associated with animals. We showed isolates of *Xiphinematobacter* conserve essential amino acid and vitamin biosynthetic pathways and yet hold increased standing genetic variation and allele fixation in some of the genes in these pathways, suggesting enhanced adaptive potential in nutrient provisioning by this symbiont. Finally, sequence divergence and phylogenetic evidence provide support for the focal isolates as a distinct species of *Xiphinematobacter* described below. Broadly, these analyses support a genomics-based approach to species designation that could be useful for other experimentally intractable insect and nematode symbiosis.

### Description of “*Candidatus* Xiphinematobacter luci” n. sp.

*Xiphinematobacter luci* (lu′ci. N.L. gen. masc. n. luci of Luc, i.e., lives in the species *Xiphinema luci*, which was named by Lamberti and Bleve-Zacheo [74] from specimens collected by Michel Luc).

Characteristics are as defined by the DNA sequence analysis description for the genus *Xiphinematobacter*, including 16S rRNA gene similarity among species (53) compared with outgroup *Verrucomicrobia*. Characteristics distinguishing this species are phylogenomic and 16S rRNA phylogeny placement in a supported monophyletic clade (phylotype K) with high 16S rRNA gene sequence similarity, 99.3%, between isolates (this study) and two related isolates of *Xiphinematobacter* sp. from *Xiphinema luci* in Spain (GenBank accession KT735064 and KT735065). This monophyletic clade is diverged in 16S rRNA gene sequences from all three formally described *Xiphinematobacter* species (53), including 4% divergence from *Xiphinematobacter rivesi* (GenBank accession AF217461), 4.6% divergence from *Xiphinematobacter americani* (AF217460), and 7.9% divergence from Xiphinematobacter brevicolli (GenBank accession AF217462). Other distinguishing characters include a distinct genomic repertoire from other *Xiphinematobacter* isolates (strains XipG or *Xiphinematobacter* sp. Idaho Grape, GenBank accession CP012665.1; and Xip2 assembled from GenBank BioSample SRX1527792) which also show 16S rRNA gene sequence divergence of 4.1%.

## MATERIALS AND METHODS

### Field sample collection and nematode isolation from soil.

To obtain new *Xiphinematobacter* isolates, approximately 100 to 500 g of soil and roots were collected from locations in Texas and New Mexico (see Table S1), using a double-serrated shovel (Root Assassin, USA) or nonspiral soil auger. Collection and transport were performed in compliance with USDA APHIS permits. Soil and roots were kept in coolers (<10°C) and processed within a few days. Nematodes were isolated by Baermann funnel and, if needed, also using sucrose flotation. Briefly, soil from each sample was divided into ∼100-g aliquots and placed in the top of glass funnels lined with two layers of Kimwipe (Kimtech, USA). Distilled water was added to saturate soil, and nematodes were collected over 3 to 5 days in clamped tubing at the bottom. Nematodes were further cleaned by three repetitions of adding 50 ml sterile distilled water, gentle centrifugation (400 × *g* for 3 min), and removal of the supernatant. After the rinse steps, nematodes were inspected in water using an inverted microscope. If soil was present, nematodes were further cleaned by mixing with 1:1 80% sucrose solution, gently centrifuging, and rinsing on a 20-μm mesh sieve (75).

### DNA extraction, Illumina library preparation, and sequencing.

To obtain and sequence symbiont genomes, DNA was isolated from bulk nematode communities (∼500 to 2,000 nematodes) by using five cycles of freeze-thaw to break cuticles, followed by DNA isolation using the Qiagen DNeasy blood and tissue kit (Valencia, CA) following the manufacturer’s directions. DNA was checked for quantity and quality on the Nanodrop spectrophotometer, and then ∼0.5 to 1 μg of DNA was used for shotgun metagenomic library preparation with the QIAseq FX 96 DNA library kit (Valencia, CA) with enzymatic fragmentation and AMPure bead size selection targets of 450 to 550 bp. Libraries were checked for quality and quantified on the TapeStation 2200 system (Agilent, USDA) before normalizing and pooling multiplex barcoded libraries. Illumina HiSeq 150-bp paired-end sequencing was performed at Genewiz, Inc. (NJ).

### Sequence assembly and assessment.

To process and assemble reads into full *Xiphinematobacter* genomes, read overlaps were merged using PEAR v0.9.11 (76). We find this step improves read assembly into contigs if performed before downstream steps. Next, merged and unmerged paired reads were filtered and trimmed using Trimmomatic v0.38 (44). Reads (trimmed merged and paired) were then assembled with metaSPAdes v3.13.0 (77, 78) using lower kmers (25, 33, and 45) and higher kmers (65, 87, 101, and 115). Initial assembly quality assessment was performed using QUAST v5.0.1 (79). To isolate all contigs matching *Xiphinematobacter* sp., blastn in BLAST+ v2.10.1 (80) (E value, 10) was used to search for matches to a custom database from the available genome for *Xiphinematobacter* sp. Idaho Grape (GenBank accession CP012665.1). The resulting contigs were extracted and then subjected to a second blastn against the nucleotide database, and all top hit contigs to *Xiphinematobacter* sp. were extracted using custom scripts. Resulting low and high kmer contigs were imported into Geneious Prime v2020.0.4 (Biomatters, Ltd.) and aligned using the ProgressiveMauve v1.1.1 (81) plugin and LASTZ alignment plugin v7.0.2 (Biomatters, Ltd.) to order and orient contigs to one another within each sample and against samples with high sequence identity, and then contigs were inspected for coverage discrepancies (if any) between contigs within a sample. Finally, similar-coverage contigs were laced together with or without regions of Ns relative to high-similarity reference contigs using the consensus among LASTZ-oriented contigs to consolidate regions of overlap between assemblies within each sample to generate long contigs (genomes). Final genome completeness was assessed based on an evaluation of annotated genomes (described below), evaluating the relative presence of housekeeping genes and tRNAs.

### Annotation, ortholog detection, pangenomes, and gene repertoire analysis.

To compare genome content functionally between *Xiphinematobacter* isolates and related *Verrucomicrobia* sp., genomes were first annotated using Prokka v1.13.3 (82) (E value, 0.001) which uses Prodigal for *ab initio* gene prediction, HMMER3 for protein family profiles, BLAST+ for comparative annotation, Barrnap for rRNAs, and Aragorn for tRNAs. For consistency among gene annotation calls, all genomes (complete or incomplete) were annotated using the same Prokka parameters. In addition to genomes from *Xiphinematobacter* isolates from Texas and New Mexico, we downloaded and annotated the genome from *Xiphinematobacter* sp. Idaho Grape (GenBank accession CP012665.1), a second variant draft genome sequence obtained from the same study, and five outgroup *Verrucomicrobia* genomes from Spartobacteria bacterium AMD-G4 (NEUK01000001.1), Chthoniobacter flavus Ellin428 (ABVL01000062.1), Terrimicrobium sacchariphilum NM-5 (NZ_BDCO01000003), “*Candidatus* Udaeobacter sp. AEW3 Udaeo2_1” (JAALOD010000001.1), and Akkermansia muciniphila YL44 (NZ_CP021420.1). Orthologs and pangenomes were obtained using Roary v3.13.0 (83), which uses blastp on the gff files produced by Prokka, using parameters -e for codon-aware alignment using PRANK (84), using -i 60 to allow for distant matches with outgroups, and using the subpackage FastTree v2.1.12 (85) with -gtr -nt < core_gene_alignment.aln > file.new to generate a preliminary tree. Using Roary’s gene_presence_absence.csv output, subsets of present/absent genes corresponding to different isolates and outgroups were extracted for the whole genome and metabolic pathway comparisons. Whole-genome presence-absence visualization was performed using Phandango (86). Pangenome overlap was visualized using InteractiVenn (87). Gene assignment to pathways was performed using a custom database based on function annotations primarily from UniProtKB downloads for available specific genomes and supplemented with pathway data from MetaCyc, KEGG pathways, and EMBL-EBI InterPro. Visualization of presence-absence within pathways was performed in R with ggplot2 “melt” and reshape2 packages.

### Phylogenetic and phylogenomic analysis.

To understand the evolutionary relationships for this symbiosis, phylogenetic analysis was performed on *Xiphinematobacter* sp. and outgroups for nearly full-length 16S rRNA genes and partial 16S rRNA genes and for *Xiphinema* spp. on partial cytochrome oxidase I (COI) genes. Sequences for these analyses were either obtained in this study or downloaded from GenBank (National Center for Biotechnology Information [NCBI]). Genes were aligned with MAFFT v1.0.4 (88). Maximum likelihood phylogenetic analysis was performed using RAxML v4.0 (89) with the GTR Gamma nucleotide model, with rate heterogeneity alpha estimated, and with rapid bootstrapping and search for the best-scoring ML tree (-f a -x 1) with 500 replicates. Bayesian inference phylogenetic analysis was performed using MrBayes v2.2.4 (90, 91) with substitution model GTR+G with 4 categories and with Markov chain Monte Carlo settings as follows: chain length 1,100,000; 4 heated chains; heated chain temperature, 0.2; subsampling frequency, 200; Burn-in length, 100,000; with random seed, 31,569; and priors with unconstrained branch lengths GammaDir (1,0.1,1,1), checking for convergence with minESS of >200. Phylogenomic analysis was performed using the set of core conserved genes from Roary core_gene_alignment.aln (nucleotides aligned by codons) for *Xiphinematobacter* sp. and outgroups. Prior to phylogenetic analysis, alignment positions with gaps in > 5% of sequences were removed. Phylogenomic analyses were performed with RAxML MrBayes as described above. Final phylogenetic trees were visualized in FigTree v1.4.4 (http://tree.bio.ed.ac.uk/software/).

### Analysis of nematode communities.

To characterize nematode communities in our genome skimming samples, blastn matches between contigs from each of the 11 sample sites to our custom nematode COI database were filtered for matches of >75% identity and 100 bp long, and they were assessed using the kmer coverage conversion to coverage equation C = (C_K_⋅R)/(R − K + 1), where C is total coverage, C_K_ is kmer coverage, K is the length of kmers, and R is read length. For hits to the same species within a sample, coverages were added (i.e., combining variants) to plot abundances of each species in a proportional bar plot. For classification of hits into *Xiphinema americanum* species complex or non-*X. americanum Xiphinema* spp., designations were gathered from published classification studies (23, 25, 35, 92, 93), and absolute coverage levels were calculated as above and plotted in a bar plot.

### SNP analysis and DBGWAS.

To determine if the gene- and pathway-specific mutations in nutritional pathways were in these symbionts, we used the output of *Xiphinematobacter* sp.-only ortholog analysis in Roary, and the core_gene_alignment.aln alignment file was analyzed to extract a variant call format (vcf) of polymorphisms (fixed SNPs within genome isolates) using snp-sites v2.5.1 (94). The number of SNPs per gene was calculated and then normalized for the gene length to obtain relative SNP densities. Within these data, gene assignment to pathways (described above), such as essential amino acid, nonessential amino acid, and vitamin/cofactor or coenzyme biosynthesis, were distinguished and plotted. Box and whisker plots were drawn, and Mann-Whitney U tests were performed in excel.

To determine if host species place differential selection on nutritional pathways in these symbionts, we used de Bruijn-based genome-wide association analysis in DBGWAS (33, 95) for whole annotated nucleotide fasta (genome) files from *Xiphinematobacter* sp. with outgroup *Verrucomicrobia* used as the input along with a phenotype file designating phenotypes as 0 = host clade *Xiphinema luci*, 1 = host clade *Xiphinema rivesi*, and 2 = host clade outgroups. Resulting DBGWAS analyses produced variant clusters or nodes (denoted DBGWAS SNPs) at various levels of significant association with phenotypes. Significant DBGWAS SNP sites distinguishing phenotypes 0 and 1, enriched for fixed variants, were extracted for gene ontology (GO) enrichment analysis (described below) and plotted as described above for total SNPs.

### GO enrichment analysis.

To determine functional overrepresentation in gene content and SNPs among *Xiphinematobacter* isolates, gene ontology (GO) enrichment analysis was performed on the *Xiphinematobacter* sp.-only pangenome not overlapping with outgroup *Verrucomicrobia*, designated “diff,” and the universe gene set included all genes in both sets. Full hierarchical GO annotations for the universe gene set were obtained primarily from UniProtKB and as needed from other databases (MetaCyc and KEGG). Enrichment analyses were performed in topGO v2.4.0 (96) which accounts for GO-term graph topology. TopGO was implemented in R using the script aip_topgo_usage.consider_universe.R (https://github.com/lyijin/topGO_pipeline/). Test statistics were assigned by the “weight01.fisher” algorithm for which returned *P* values are regarded as corrected (or not affected) by multiple testing. GO enrichment analysis in topGO was also performed on the genes with statistically significant DBGWAS SNPs associated with host *Xiphinema* phenotype as the filter on the complete *Xiphinematobacter* diff gene set in topGO.

### Analysis of dN/dS and site-specific positive selection.

To assess differential selection pressures on proposed symbiont-supplementation pathways, we analyzed within and between *Xiphinematobacter* species signatures of selection based on statistically significant GO biological process terms for nutritional pathways, beginning with the Roary core_gene_alignment.aln alignment file with the corresponding embl headers imported into Geneious. Pathway-associated gene alignment blocks were extracted and concatenated from the full *Xiphinematobacter* alignment. From each pathway subalignment block (e.g., for each set of genes associated with an amino acid or vitamin/cofactor biosynthetic pathway identified in DBGWAS) as well as for the remaining genome, dN/dS was calculated using the Nei and Gojobori 1986 estimator within PAML 4 CodeML (97), implemented through EasyCodeML v1.21 (98). For the same alignment blocks, we used CodeML with the Site Model analysis variance across the sequence blocks by applying the likelihood ratio test (LRT) for positive selection (*ω*), in which multiple nested models were evaluated. In particular, the LRT tests for selecting M8 over M7 [M7 beta; *ω ∼ B(p*,*q)* with 2 free parameters, and 10 categories or equal classes; versus M8 beta& *ω*; with proportion p0 of sites ∼B(p,q),p1 of sites from discrete *ω* class with 4 free parameters, and 11 categories] which has been shown to be a very stringent test of positive selection (97–99).

### Data availability.

The raw SRA data are deposited in GenBank under BioProject accession number PRJNA687334, and the genomes are in GenBank under BioSample accession numbers SAMN17141338, SAMN17141340, SAMN17141343, SAMN17141344, SAMN17141345, and SAMN17141353.
