# Supplementary material for: Essential Amino Acid Enrichment and Positive Selection Highlight Endosymbiont's Role in a Global Virus-Vectoring Pest
Source: mSystems. 2021 Feb 2;6(1):e01048-20. doi: 10.1128/mSystems.01048-20 (PMC7857533; doi:10.1128/mSystems.01048-20)
Supplement: TABLE S1 [file mSystems.01048-20-st001.docx]

| Sample Name | Plant Host | Location | Lat/Long |
| --- | --- | --- | --- |
| P15 * | Citrus (farm) | Los Fresnos, TX | 26.132554 / -97.473898 |
| P18 * | Wild grasses (roadside) | Los Fresnos, TX | 26.125003 / -97.395915 |
| P19 | Wild grasses (roadside) | La Prior, TX | 29.662474 / -95.376874 |
| P21 * | Wild grasses (roadside) | Junction, TX | 30.479468 / -99.778204 |
| P22 * | Wild shrubs (roadside) | Carlsbad, NM | 32.438692 / -104.178553 |
| P23 * | Wild shrubs (roadside) | Laredo, TX | 27.535932 / -99.433883 |
| P24 | Olive trees (farm) | Weslaco, TX | 26.167820 / -98.010570 |
| P29 | Mixed vegetables (farm) | Lubbock, TX | 33.517183 / -101.811984 |
| P3-11 * | Fruit trees (farm) | Los Fresnos, TX | 26.158570 / -97.384450 |
| P31 | Wild grass roots (roadside) | Lampasas, TX | 30.962257 / -98.052659 |
| P32 | Wild grass soil (roadside) | Lampasas, TX | 30.962257 / -98.052659 |
