# Supplementary material for: Essential Amino Acid Enrichment and Positive Selection Highlight Endosymbiont's Role in a Global Virus-Vectoring Pest
Source: mSystems. 2021 Feb 2;6(1):e01048-20. doi: 10.1128/mSystems.01048-20 (PMC7857533; doi:10.1128/mSystems.01048-20)
Supplement: TABLE S2 [file mSystems.01048-20-st002.docx]

| Sample Name | # of Reads  (millions) | # of *Xiphinema* spp. | *Xiphinematobacter* only: | | | |
| --- | --- | --- | --- | --- | --- | --- |
|  |  |  | # of Contigs  (low; high kmer) | Length of Maximum Contig (low kmer; high kmer) | N50  (best) | Cov. of Maximum Contig |
| P15 * | 284.6 | 5 | 44; 122 | 871,420; 278,893 | 871,420 | 84.22 |
| P18 * | 238.2 | 8 | 46; 130 | 254,180; 47,398 | 182,636 | 28.65 |
| P19 | 69.3 | 5 |  |  |  |  |
| P21 * | 106.7 | 8 | 35; 67 | 642,083; 732,992 | 732,992 | 43.70 |
| P22 * | 170.8 | 7 | 115; 421 | 40,066; 74,973 | 16,926 | 23.45 |
| P23 * | 139.5 | 5 | 38; 77 | 501,379; 54,867 | 350,397 | 47.05 |
| P24 | 61.8 | 2 |  |  |  |  |
| P29 | 123.9 | 5 |  |  |  |  |
| P3-11 * | 704.8 | 9 | 474; 1052 | 429,999; 569,901 | 217,493 | 25.77 |
| P31 | 60.7 | 3 |  |  |  |  |
| P32 | 249.0 | 6 |  |  |  |  |
